# Precursor-Dependent Photocatalytic Activity of Carbon Dots

**DOI:** 10.3390/molecules25010101

**Published:** 2019-12-26

**Authors:** Amadio Emanuele, Simone Cailotto, Carlotta Campalani, Lorenzo Branzi, Carlotta Raviola, Davide Ravelli, Elti Cattaruzza, Enrico Trave, Alvise Benedetti, Maurizio Selva, Alvise Perosa

**Affiliations:** 1 Department of Molecular Sciences and Nanosystems, Ca’ Foscari University of Venice, 30172 Venezia Mestre, Italy; simone.cailotto@unive.it (S.C.); carlotta.campalani@unive.it (C.C.); lorenzo.branzi@unive.it (L.B.); cattaruz@unive.it (E.C.); enrico.trave@unive.it (E.T.); benedett@unive.it (A.B.); selva@unive.it (M.S.); alvise@unive.it (A.P.); 2 PhotoGreen Lab, Department of Chemistry, University of Pavia, 27100 Pavia, Italy; carlotta.raviola01@universitadipavia.it (C.R.); davide.ravelli@unipv.it (D.R.)

**Keywords:** carbon dots, photocatalysis, carbon source, biomass valorization

## Abstract

This work systematically compares both structural features and photocatalytic performance of a series of graphitic and amorphous carbon dots (CDs) prepared in a bottom-up manner from fructose, glucose, and citric acid. We demonstrate that the carbon source and synthetic procedures diversely affect the structural and optical properties of the CDs, which in turn unpredictably influence their photo electron transfer ability. The latter was evaluated by studying the photo-reduction of methyl viologen. Overall, citric acid-CDs were found to provide the best photocatalytic performance followed by fructose- and glucose-CDs. However, while the graphitization of glucose- and citric acid-CDs favored the photo-reaction, a reverse structure–activity dependence was observed for fructose-CDs due to the formation of a large graphitic-like supramolecular assembly. This study highlights the complexity to design in advance photo-active bio-based carbon nanomaterials.

## 1. Introduction

Fluorescent carbon dots (CDs) have drawn considerable attention in a wide range of applications ranging from the biomedical [1,2,3,4,5] to energy-related [6,7] fields. Among them, CDs appeared to be a promising luminescent bio-based nanomaterial for photocatalytic applications. To date, the photocatalytic activity of CDs has been under-explored despite their remarkable light harvesting properties and excellent electron donor/acceptor capabilities. Moreover, most reported photo-systems of CDs are limited by the co-presence of precious metal complexes or enzymes as redox mediators [8,9,10,11,12]. The accurate design of CDs with tunable photo–electric properties is one of the major issues to face to achieve a real breakthrough in this field.

The luminescence properties of CDs are known to be structurally-dependent [13,14,15,16]. For instance, high photoluminescence properties were observed in amorphous CDs—owing to the presence of molecular-like fluorophores and heteroatom doping agents in their structures—synthesized under mild hydrothermal conditions [7,8,9,10,11,12,13,14,15,16,17,18,19], while graphitic-like CDs obtained via harsh pyrolytic treatments gave low luminescence. The graphitic core, indeed, acts as a quencher of the molecular fluorophore-derived photoluminescence via a fluorescence resonance energy transfer (FRET) mechanism [20].

Nonetheless, the degree of carbonization and the actual structure of CDs influence their photocatalytic behavior as well. In the photo-evolution of H_2_ through water splitting, different performances were observed using CDs—prepared from citric acid or aspartic acid—with different amorphous, graphitic, or graphitic nitrogen structures [10]. Moreover, the photoreduction of methyl viologen (MV^2+^) turned out to be affected by the quinonic precursors employed in the multicomponent synthesis of amorphous nitrogen doped CDs [18]. In all these cases, a general and unique structure–photoactivity correlation could not be exhaustively highlighted due to the high variability of the synthetic methods, carbon sources, and dopants employed in the synthesis of these nanomaterials.

In connection to the wide-ranging interests of our group in developing biomass-derived platform chemicals [21,22,23,24,25,26], we recently reported a fundamental study on the photoreduction of methyl viologen using a set of citric acid-derived CDs, paving the way towards the rational design of carbon-nanoparticles for efficient photocatalytic organic transformations [27]. These studies demonstrated that the carbon–nitrogen source, the synthetic method, and the resulting structural properties strongly affect the electrochemical properties of CDs and thus their photo-reactivity. In general, the presence of nitrogen in the amorphous structure of CDs favors the photogeneration of reactive electrons, thus improving the photoreduction efficiency.

Herein, we propose to evaluate whether the carbon sources (citric acid, glucose, or fructose) influence structural features and photocatalytic efficiency. The aim is to explore new synthetic options to design CDs for photocatalytic applications. We hereby demonstrate that the carbon source, as well as the synthetic methodologies employed, strongly affect structural and optical properties of the CDs, thus resulting in different photocatalytic activities.

## 2. Results

### 2.1. Synthesis and Characterization of the CDs

In this work, six different sets of CDs were synthetized using two different preparation methods—hydrothermal and pyrolytic, which yield amorphous (a-) and graphitic (g-) CDs, respectively—and three sets of reagents: citric acid (Cit-), glucose (Glu-), and fructose (Fru-) (see Supplementary Materials for full synthesis details). Briefly, a-Cit-CDs, a-Glu-CDs, and a-Fru-CDs were hydrothermally synthesized, as previously reported [2,27], from glucose, fructose, and citric acid, respectively, to yield amorphous nanomaterial composed of light molecular-like organic compounds as well as poorly-defined carbonaceous substances. Graphitic g-Glu-CDs and g-Fru-CDs were synthesized for the first time via harsh thermolysis of neat glucose or fructose, respectively, following the already published procedure optimized for citric acid derived g-Cit-CDs [27]. The silent ^1^H and ^13^C{^1^H}-NMR (Supplementary Materials Figure S1 and Figure S2 for g-Glu-CDs and g-Fru-CDs, respectively, and [24] for g-Cit-CDs) likely excluded the presence of molecular species and suggested the presence of NMR-inactive nano-carbonaceous solids.

After having synthesized the nanomaterials, their morphologies and optical properties were fully compared by high-resolution transmission electron microscopy (HR-TEM), scanning electron microscopy (SEM), X-ray photoelectron spectroscopy (XPS), Fourier transform infrared spectroscopy (FT–IR), photoluminescence (PL), ultraviolet−visible spectroscopy (UV−Vis), and time-resolved PL measurements.

**Figure 1:**
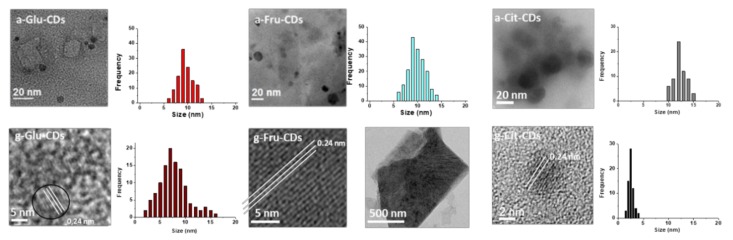
HR-TEM images of the amorphous and graphitic carbon dots (CDs).

**Figure 2:**
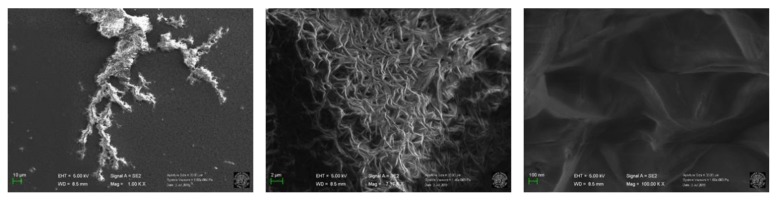
SEM images at increasing magnifications of g-Fru-CDs.

HR-TEM images (Figure 1) clearly demonstrate how the synthetic procedures affect the nanostructures: hydrothermal synthesis yielded low density and amorphous CDs, while pyrolysis rendered graphitic structures with well-defined shapes. a-Glu-CDs, a-Fru-CDs, and a-Cit-CDs were all poorly defined nanomaterials with very rarefied and irregular structures having a dimension ranging from 9 to 12 nm [2,27]. On the other hand, g-Glu-CDs and g-Cit-CDs [27] were well-dispersed nanoparticles with a quasi-spherical shape about 7–9 and 2−7 nm in size. Surprisingly, both HR-TEM (Figure 1 and Supplementary Materials Figure S3 and Figure S4) and SEM (Figure 2, Supplementary Materials Figures S5–S8) images revealed the formation only for the g-Fru-CDs of supramolecular agglomerates of graphene-like crystalline sheets with multilayers nature having dimension between 0.5 μm and 1.5 μm.

Substrate thermal decomposition influenced the structural differences of the graphitic CDs. By following the reactivity with ^1^H-NMR under pyrolysis condition (see Supplementary Materials for more details), citric acid and glucose were not fully converted after 60 min of heating while fructose quickly decomposed, leading to a silent spectrum as evidence of the reasonable formation of NMR inactive nano-structures. Citric acid decomposition pathways follow a known scheme, consisting first of a breakdown into small organic molecules (citraconic and itaconic anhydride) and then complete decomposition within 4 hours [27]. The monosaccharide sugars fructose and glucose follow instead different pathways: formic acid was mainly revealed during the thermal decomposition of fructose while acetic acid was detected from glucose [28]. Therefore, the final morphologies of the CDs may to a certain extent be derived by different decomposition pathways. Reasonably, the higher the reactivity of the carbon precursors, the easier and larger the formation of the nanoparticles.

**Figure 3:**
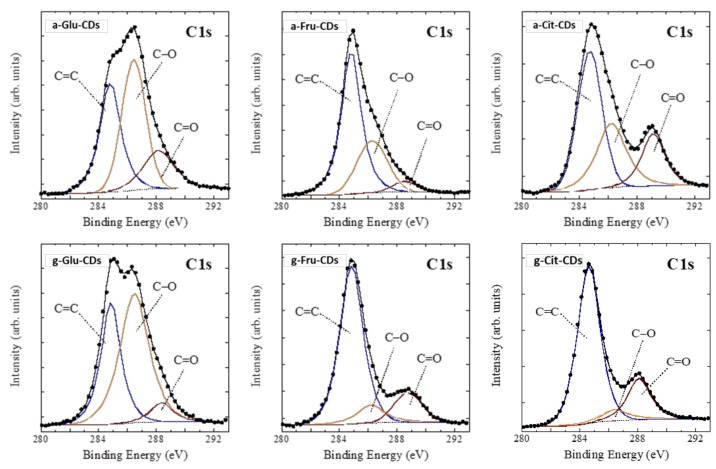
C1 XPS spectra of all the CDs samples. The binding energy (BE) was corrected for surface charging.

Further evidence of the structural/compositional differences of the CDs was given by the XPS analysis. As shown in Figure 3, all samples displayed the presence of the three strong peaks in the C1 bands at 284.6, 286.2, and 288.6 eV assigned to C=C, C‒O, and C=O functional groups, respectively, with, however, some differences. The C=C signal was predominant for citric- and fructose-CDs while C‒O was for glucose-CDs. Furthermore, the C–O band intensity, i.e., the oxygen content, markedly decreased after graphitization of citric acid and fructose as evidence of the formation of crystalline structures with predominantly graphitic sp^2^ carbon with a lower—with respect to the analogous glucose-like CDs—content of superficial carboxyl moieties.

However, no substantial differences were revealed by FT–IR analyses (Supplementary Materials Figure S12) since carboxylates—as primary surface CD functionalities—along with the formation of C(sp^2^) containing moieties were revealed in all cases. Indeed, a,g-Glu-CDs, a,g-Fru-CDs, and a,g-Cit-CDs all showed strong broad absorptions in the region 3500–3000 cm^−1^, weak signals at 2900–2800 cm^−1^, and additional stretching bands in the range of 1800–1600 cm^−1^, 1600–1400 cm^−1^, and around 1200 cm^−1^, associated with the presence of O−H, C−H, C=O, C=C, and C–O–C functional groups, respectively.

**Figure 4:**
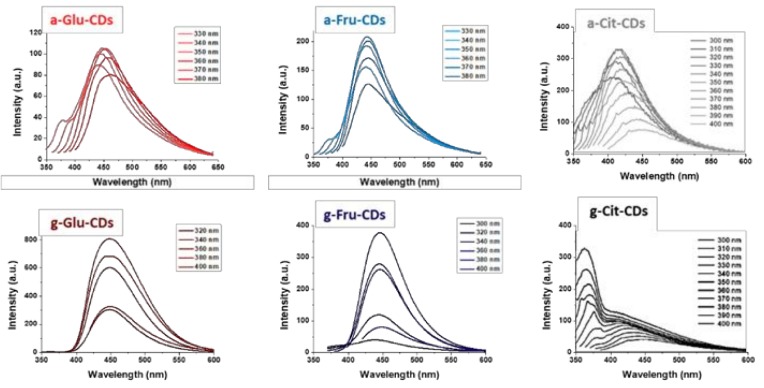
Photoluminescence (PL) spectra of amorphous and graphitic CDs.

The photoluminescence (PL) of prepared CDs (Figure 4) showed a quasi-excitation-independent emission behavior with an emission peak centered at 450 nm that could be observed for both glucose- and fructose-CDs. The citric acid-derived nanomaterials exhibited instead a complex mixture of excitation dependent emission bands whose maximum ranged between 420 and 500 nm. For g-Cit-CDs, an additional sharp band at 385 nm—slightly influenced by the excitation wavelength—was furthermore noticed due to the crystalline nano-core. The respective excitation spectra registered at different emission wavelength are shown in Supplementary Materials Figure S14.

**Figure 5:**
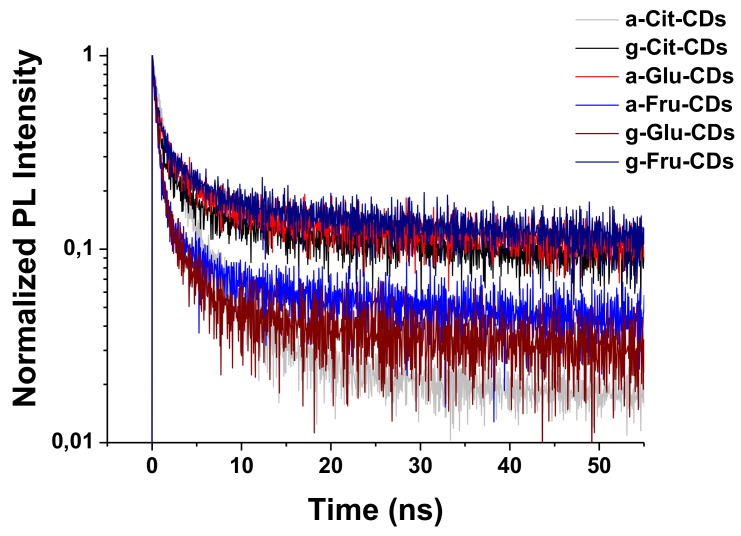
Time-resolved photoluminescent (PL) measurements of CDs.

Lastly, UV–Vis spectra of all CDs (Supplementary Materials Figure S13) exhibited absorption bands across the near-UV and tailing in the visible region. In detail, g-Cit-CDs showed relatively simple spectra having a weak absorption band at 365 nm, ascribed to n–π* transition due to defects states [27], and a strong one at 220 nm due to the π–π* transition of the aromatic domains. a,g-Fru-CDs and a,g-Glu-CDs and a-Cit-CDs revealed instead the presence of three defined absorption regions at 220–225, 260–280, and 365–370 nm associated with the π–π* transition of sp^2^ C=C conjugated systems, n–π* transitions of carbonyl groups, and surface state transitions, respectively. The photoluminescence quantum yields (QYs, based on quinine sulphate) of a-Cit-CDs and g-Cit-CDs were 1.0% and 1.2%, respectively, and a-Glu-CDs and g-Glu-CDs gave slightly higher values of 1.8% and 2.3%, respectively. Low QY values of 0.3% and 0.7%, respectively, were instead measured for a-Fru-CDs and g-Fru-CDs [2,27]. The mass extinction coefficients (ε) for CDs at 365 nm are reported in Table 1. Both g-Fru-CDs and g-Cit-CDs with graphitic cores showed significantly increased absorption (1.2 and 4 times, respectively) in comparison to the corresponding amorphous a-Fru-CDs and a-Cit-CDs. Conversely, a-Glu-CDs exhibited an improved absorption over graphitic ones (up to 2.7 times). Similar behavior was observed for the lifetimes of excited states (Table 1); the graphitic domain of g-Cit-CDs and g-Fru-CDs resulted in longer lifetimes in comparison with the analogous amorphous (from 3.3 and 4.4 for the amorphous to 5.4 and 5.9 ns for the graphitic, respectively). On the other hand, g-Glu-CDs (2.4 ns) possessed a lower lifetime compare to a-Glu-CDs (4.6 ns). All the CDs exhibited a PL decay characterized by a multiexponential behavior, whose parameters are reported in Supplementary Materials Table S1 and Figure 5.

### 2.2. Photocatalytic Experiment

To investigate the relationship between the structural features and their photo-activity, all CDs were subsequently employed as photo-redox catalysts in the single electron photoreduction of methyl viologen MV^2+^ (−0.45 V vs. NHE) [29] to its mono-reduced species (MV^•+^). The photoreductions were carried out in aqueous media in the presence of ethylenediaminetetraacetate (EDTA) as a sacrificial electron donor and under LED light irradiation (365 nm) using a concentration of each CD normalized for absorption (0.5 a.u.). The lack of activity in the absence of light and CDs (Figure S15) indicated the primary role of the photo-excited CD-states in the photoinduced electron transfer (PET) reactivity.

**Figure 6:**
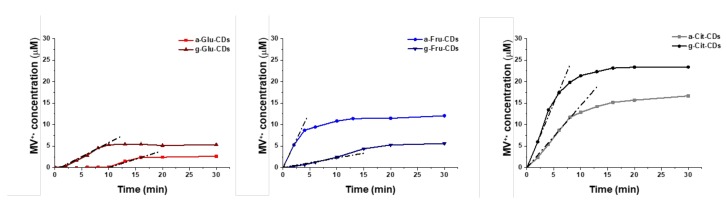
Reaction kinetics of formation of MV^•+^ using CDs as photo-redox catalysts. Reaction conditions: MV^2+^ 60 μM, EDTA 0.1 M, λ_exc_: 365 nm with an absorbance-normalized amount of CDs and water as solvent (see Supplementary Materials for more details).

As shown in Figure 6 and Table 2, the PET ability was found to be strongly dependent on the carbon precursor. Citric acid-derived CDs were the most photoactive nano-systems, followed by fructose and glucose analogues. The amorphous or graphitic structures influenced CD reactivity, further supporting the unpredictability of CD photochemical behavior. In this case, the graphitization of citric acid and, surprisingly, also glucose-derived CDs enhanced their photocatalytic performance ca. 1.5 and 1.6 times, respectively, from an initial rate of 3.45 to 5.06 × 10^−8^ M × s^−1^ for a,g-Cit-CDs and from 0.65 to 1.07 × 10^−8^ M × s^−1^ for a,g-Glu-CDs (Table 2 entries 5,6, and 1,2). Nonetheless, a-Glu-CDs also exhibited an induction time of 10 min. The opposite trend was instead observed for a,g-Fru-CDs, with the amorphous material being 6.7 time more reactive—from an initial rate of 4.37 to 0.67 × 10^−8^ M × s^−1^—than the graphitic counterpart (Table 2 entries 3,4). The reason for this inversion was that the reactive performances reasonably rely on the formation of the large graphitic materials and supramolecular aggregates, which may affect the PET efficiency. Overall, the results herein reported highlight the complexity in assessing PET and in designing CDs-based photocatalytic organic applications.

## 3. Conclusions

In conclusion, the photocatalytic properties of monosaccharide-derived CDs were tested in the single electron transfer reaction towards MV^2+^. We demonstrate how the choice of the carbon precursors and its morphology can significantly influence the photo-redox behavior of the resulting materials.

The first significant finding herein described is that the hydrothermal treatment of citric acid, glucose, or fructose produced amorphous nanoaggregates containing molecular-like compounds, whereas pyrolysis formed extended graphene-like carbonaceous cores. Moreover, it was demonstrated that the higher the reactivity towards the decomposition/transformation of the carbon precursors, the easier and larger the formation of the nanoparticles was. In this regard, g-Fru-CDs resulted as a supramolecular agglomerate of graphene-like crystalline sheets with a multilayered nature.

It was also shown that synthetic methods and starting precursors strictly affected the photo and optical properties of the CDs as well. Citric acid, as a carbon source, yielded the best performing photoactive nanomaterials followed by fructose and glucose derivatives. Another important finding relied on the effect of the graphitization of the carbon core of CDs on their PET activity. The morphology of the carbon-dots induced either a positive or negative photo-reactivity trend, which in turn was influenced by the carbon-precursor employed. For fructose-CDs, the amorphous structure provided optimal results in terms of initial rates; likewise, the graphitic nanoparticles were more active when formed by citric acid and glucose.

Overall, these results provide important insights into carbon source/structure/activity relationships and may pave the way for a more rational development of carbon-derived nano-photocatalysts for organic synthesis.

## Figures and Tables

**Table 1:** Mass extinction coefficients (ε) for CDs calculated at 365 nm and lifetime of the excited states (τ).

Entry	ε (L × g−^1^ × cm^−1^)	τ (ns)
a-Glu-CDs	1.71	4.6
g-Glu-CDs	0.63	2.4
a-Fru-CDs	1.63	4.4
g-Fru-CDs	2.01	5.9
a-Cit-CDs	1.16 [27]	3.3
g-Cit-CDs	4.68 [27]	5.4

**Table 2:** Photocatalytic performance of the tested CDs.

Entry	CDs	ν_0_ × 10^−8^ (M × s^−1^)	Relative Rate
1	a-Glu-CDs	0.65 ^a^	1
2	g-Glu-CDs	1.07	1.6
3	a-Fru-CDs	4.37	6.7
4	g-Fru-CDs	0.67	1
5	a-Cit-CDs	3.45 [27]	5.3
6	g-Cit-CDs	5.06 [27]	7.8

For reaction conditions, see Figure 6a. Calculated without considering the induction time.

## Supplementary Materials

The following are available online at https://www.mdpi.com/1420-3049/25/1/101/s1: Materials and methods, synthesis and characterization of CDs, thermal decomposition studies, TEM and SEM images, XPS, FT–IR, UV spectra, time-resolved photoluminescence, and photocatalytic experiments.
