# Supplementary material for: Precursor-Dependent Photocatalytic Activity of Carbon Dots
Source: Molecules. 2019 Dec 26;25(1):101. doi: 10.3390/molecules25010101 (PMC6983025; doi:10.3390/molecules25010101)
Supplement: Supplementary file 1 [file molecules-25-00101-s001.pdf]

Supporting Information

Communication

Precursors-Dependent Photocatalytic Activity of Carbon Dots

Emanuele Amadio*,[1], Simone Cailotto[1], Carlotta Campalani[1], Lorenzo Branzi[1], Carlotta Raviola,[2] Davide Ravelli[2], Elti Cattaruzza[1], Enrico Trave[1], Alvise Benedetti[1], Maurizio Selva[1], Alvise Perosa[1]

1 Department of Molecular Sciences and Nanosystems, Università Ca' Foscari Venezia, Via Torino 155, 30172 Venezia Mestre, Italy;

2 PhotoGreen Lab, Department of Chemistry, University of Pavia, viale Taramelli 12, 27100 Pavia, Italy

* Correspondence: emanuele.amadio@unive.it;

Received: date; Accepted: date; Published: date

Table of Content

1.1. General Information 2
1.2. Synthesis and characterization of a,g-Cit-CDs, a,g-Glu-CDs and a,g-Fru-CDs 2
1.3. TEM images of g-Fru-CDs 4
1.4. SEM images of CDs 6
1.5. Thermal decomposition of citric acid, glucose and fructose 8
1.6. XPS analysis 10
1.7. FT-IR Spectra of CDs 10
1.8. UV-Vis spectra 10
1.9. Time-resolved photoluminescence (PL) measurements 11
1.10. Photocatalytic experiments 12
1.11. References 13

1.1. General Information

Glucose, Fructose, sodium hydroxide (NaOH) and Methyl viologen (MV^{2+}) were purchased from Sigma-Aldrich and used as received without further purification. MilliQ water was employed in all the experiments.

^{1}H, $^{13}C\{^{1}H\}$, DOSY NMR spectra were recorded on a Bruker AV 300 (^{1}H: 300 MHz; ^{13}C: 75.5 MHz) spectrometer. For ^{1}H and $^{13}C\{^{1}H\}$ NMR the chemical shifts (δ) have been reported in parts per million (ppm) relative to the solvent signal as an internal reference. UV/Vis absorption spectra were recorded by using an Agilent 8456 spectrophotometer. Photoluminescence (PL) experiments were performed with a Perkin Elmer LS 55 fluorescence spectrophotometer. The Fourier transform infrared spectroscopy (FT-IR) was measured at wavenumbers ranging from 400 to 4000 cm^{-1} using a Perkin-Elmer spectrum one FT-IR spectrometer. The morphologies of the resulting CDs were analyzed by a JEM-2200FS transmission electron microscope (TEM) operating at 200 kV.

1.2. Synthesis and characterization of a,g-Cit-CDs, a,g-Glu-CDs and a,g-Fru-CDs

The nanomaterials were obtained as follow:

1. amorphous citric acid CDs (**a-Cit-CDs**) were hydrothermally synthesized, as already reported,[1] by heating an aqueous solution of citric acid in a sealed autoclave for 24 h at 180 °C. The mixture was then neutralized to pH 7 with aqueous NaOH and evaporated to dryness leading to a dark yellow luminescent oil ($25\%_{wt}$ yield) which was used without any further purifications.

2. graphitic citric acid CDs (**g-Cit-CDs**) were synthetized, as already reported,[1] by pyrolysis heating neat citric acid under air at 220 °C for 48 h. The resulting solid was then neutralized to pH 7 with aqueous NaOH, and the crude mixture dialyzed (cut-off Mn: 1.0 kDa) for 24 h against fresh water. The inner solution was evaporated to dryness leading to a dark-black solid ($20\%_{wt}$ yield).

3. amorphous glucose and fructose CDs (**a-Glu-CDs** and **a-Fru-CDs**) were hydrothermally synthesized, as already reported,[2] by heating an aqueous solution of glucose or fructose in a sealed autoclave for 24 h at 200 °C. The mixtures were centrifugated to remove the black precipitate, the supernatant was then filtered and evaporated to dryness leading to a dark solid ($34\%_{wt}$ yield and $29\%_{wt}$ for glucose and fructose respectively) which were used without any further purifications.

4. graphitic glucose and fructose CDs (**g-Glu-CDs** and **g-Fru-CDs**) were synthetized by a pyrolysis treatment of 100 g of glucose or fructose, respectively, in a conical flask at 220 °C for 48 h under air. As the reaction proceeds, the solution turns from colorless to orange and, in the end, to dark brown. The as formed suspension was cooled at *r.t.*, neutralized to pH 7 by the addition of NaOH 5M and the resulting solution was then dried leading to a dark brown solid. The crude was dissolved in milliQ water and dialyzed (cutoff Mn: 1.0 kDa) for 24 h against fresh water, that was refreshed after 12 hours, to isolate the pure carbogenic core from the residual volatile NMR-active molecules, yielding $65\%_{wt}$ and $48\%_{wt}$ of **g-Glu-CDs** and **g-Fru-CDs** after freeze-drying. In Figure S1 and S2 the ^{1}H and $^{13}C\{^{1}H\}$ NMR comparison between reagents and final purified nanoparticles are shown.

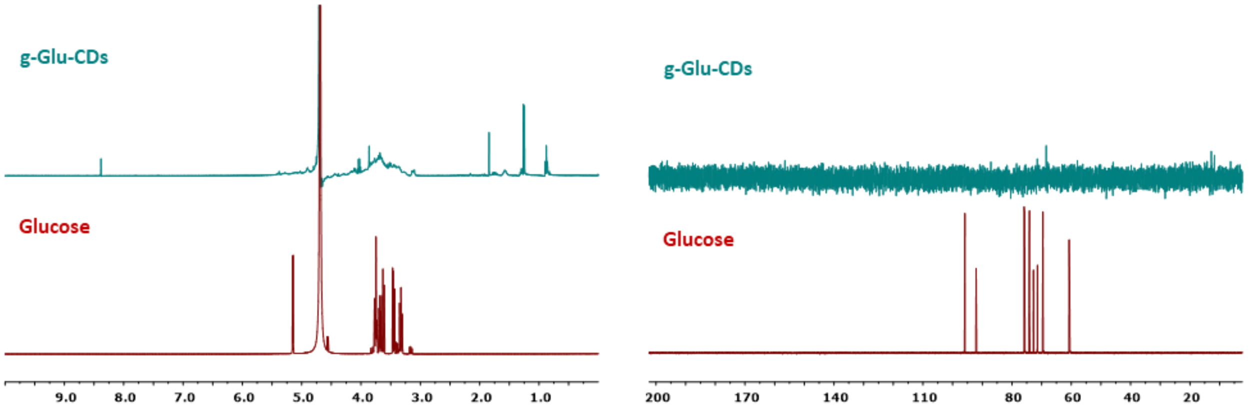

Figure S1. ^{1}H-NMR and ^{13}C-NMR in D_2O of **g-Glu-CDs** compared with glucose.

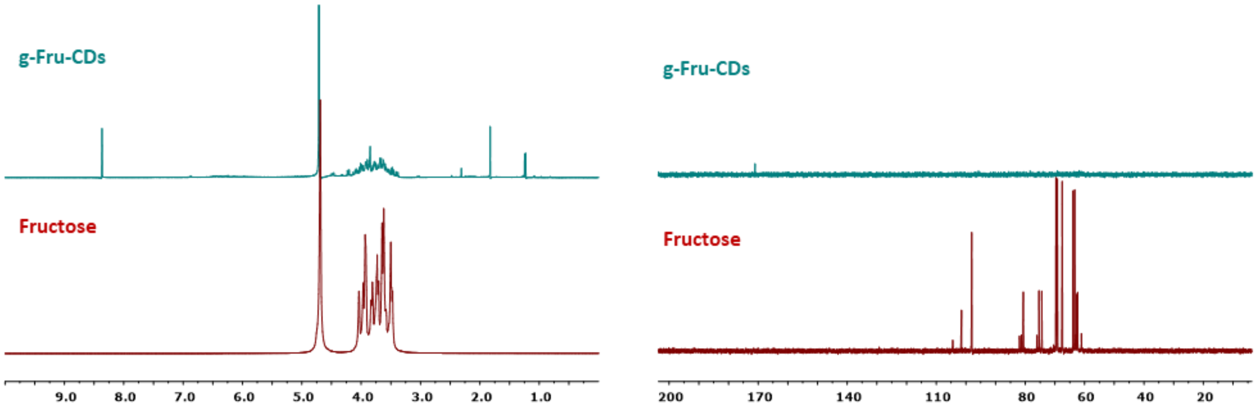

Figure S2. ^{1}H-NMR and ^{13}C-NMR in D_2O of **g-Fru-CDs** compared with fructose.

1.3. TEM images of g-Fru-CDs

Measurement details: High resolution electron microscopy images were recorder using FEI TALOS F2005 (voltage 200 kV). The sample was prepared by dispersion of the powder in bi-distilled water (concentration around 1 mg/ml), the as prepared solution was sonicated for 20 min to increase the dispersion of the material. One drop of solution was deposited onto a copper grid and dried at ambient temperature before the analysis.

The TEM analysis of the **g-Fru-CDs** revealed the presence of extended layer structure with a wide range of dimensions, most of them with dimension between 0.5 µm to 1.5 µm (Figure S3). Furthermore, the SAED pattern reveals single crystalline nature of the multilayer system with the expected six-fold symmetry.[3,4,5] The HRTEM analysis of areas where more layers are superimposed reveals the presence of extended crystalline areas (Figure S4).

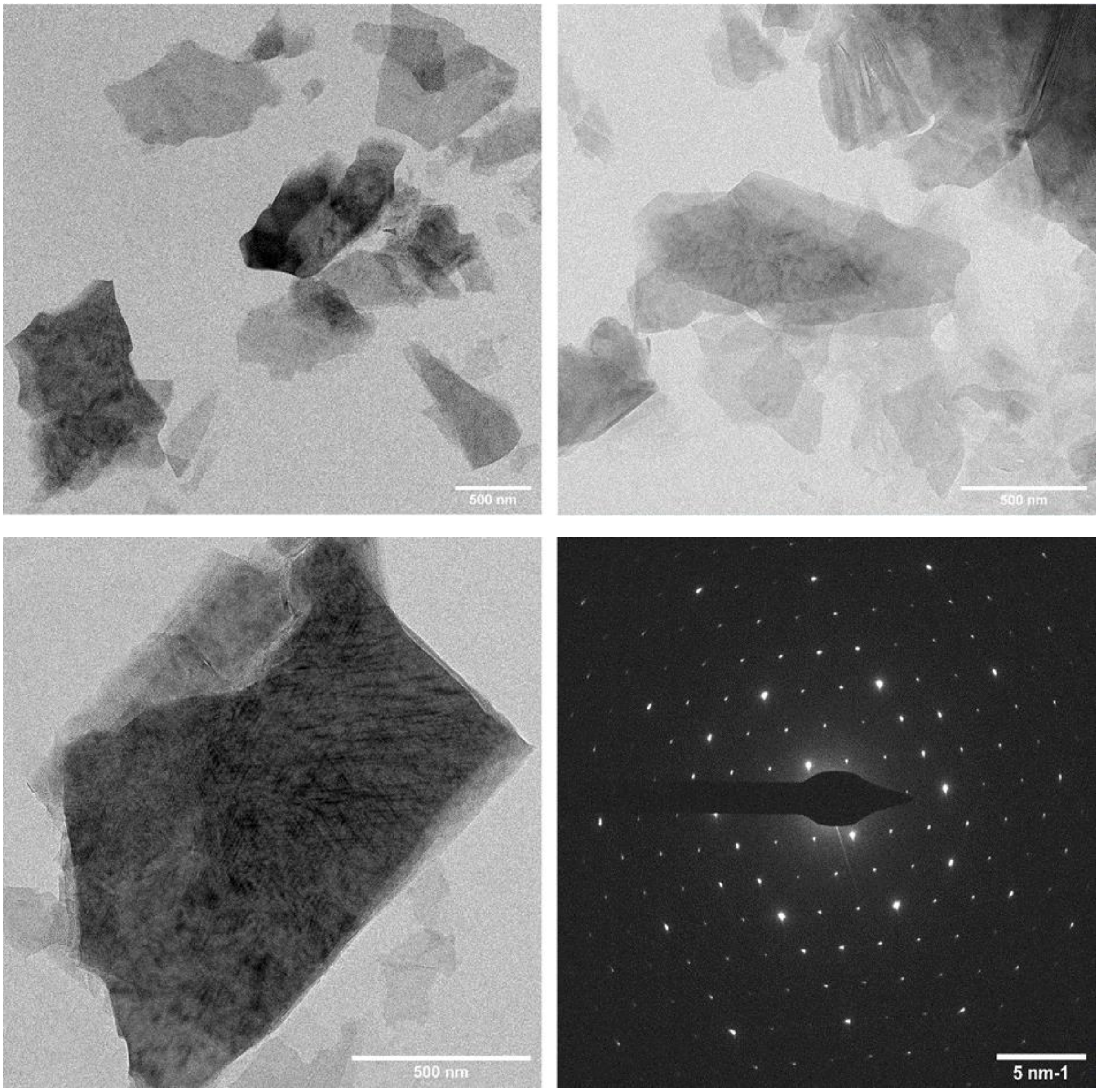

Figure S3. TEM of different areas of the sample **g-Fru-CDs** and SAED pattern.

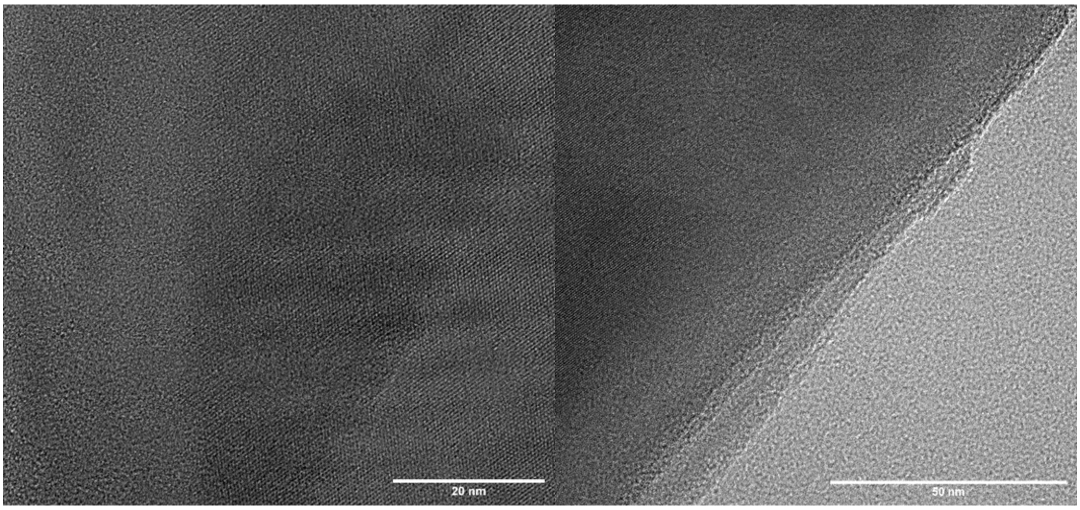

Figure S4. Extended HRTEM analysis.

1.4. SEM images of CDs

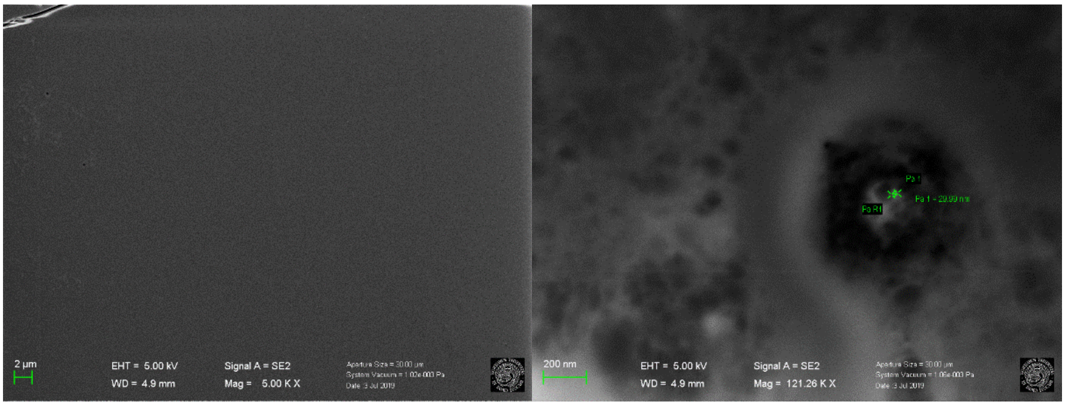

Figure S5. SEM images of **a-Fru-CDs**.

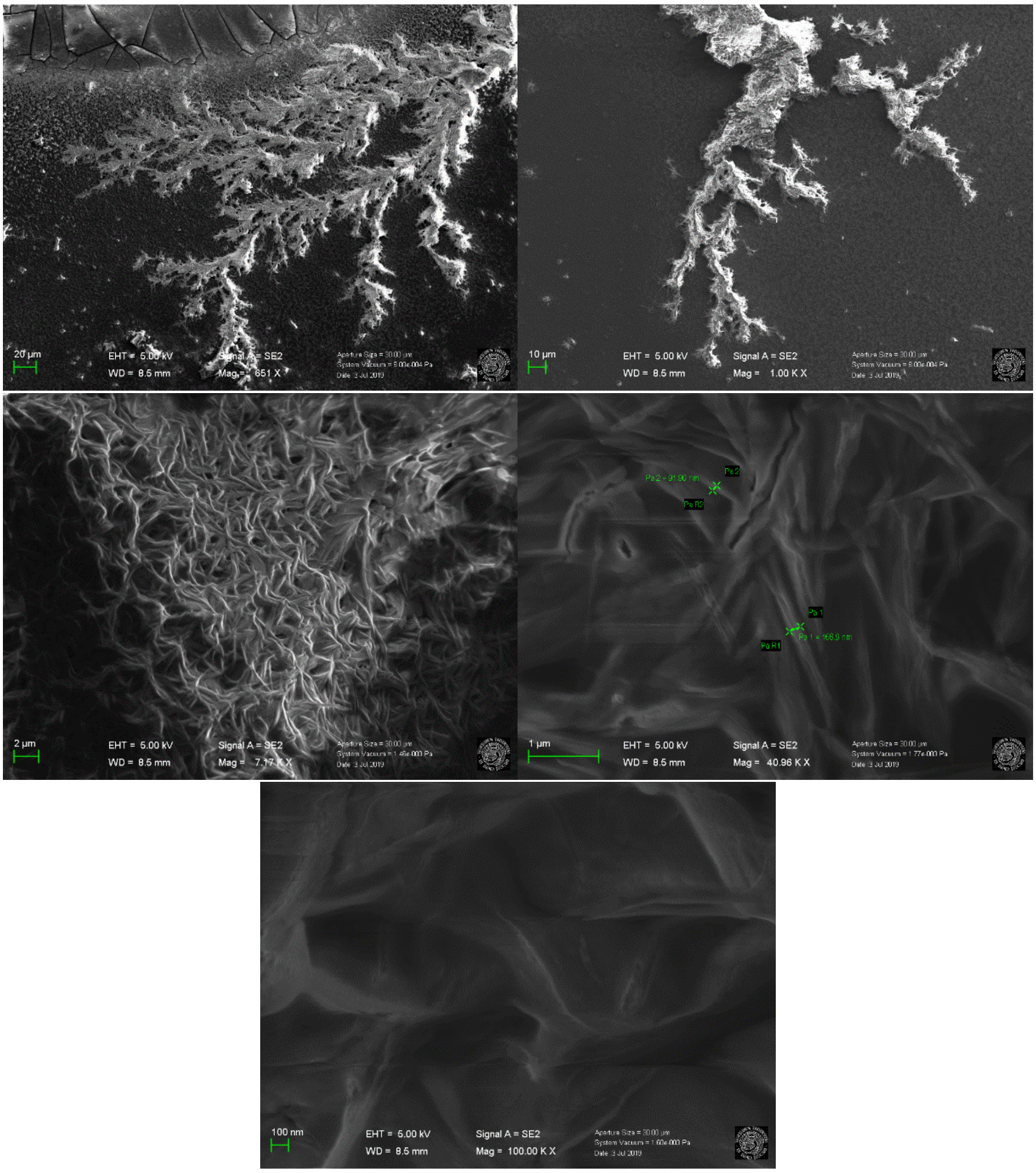

Figure S6. SEM images of **g-Fru-CDs**.

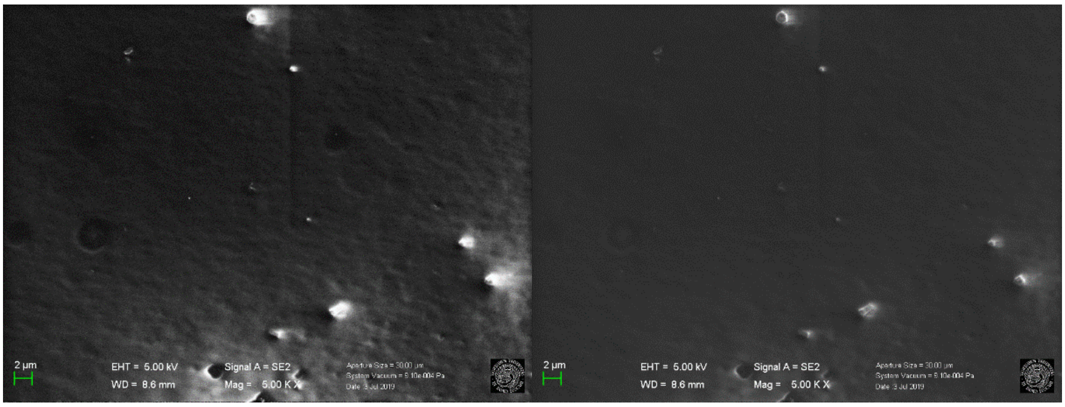

Figure S7. SEM images of **a-Glu-CDs**.

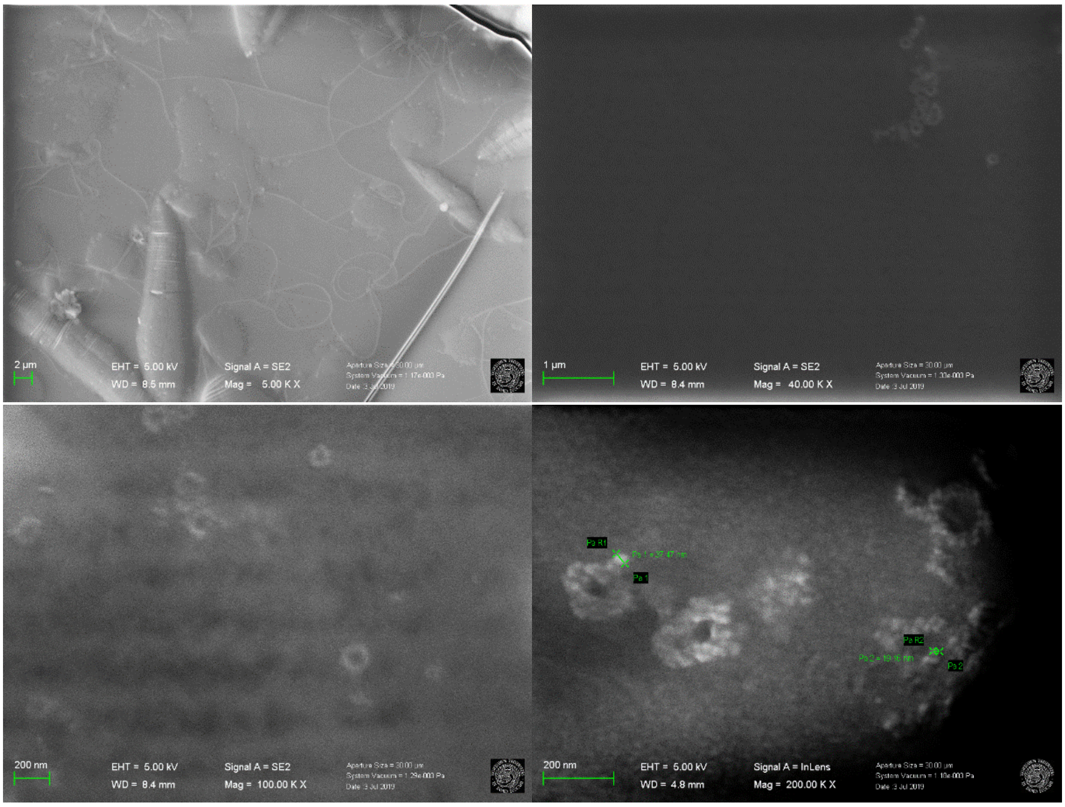

Figure S8. SEM images of **g-Glu-CDs**.

1.5. Thermal decomposition of citric acid, glucose and fructose.

1 g of citric acid, glucose or fructose were heated in a conical flask at 220 °C and the decoposition kinetics monitored *via* ^{1}H-NMR spectroscopy within 4 hours of reaction. As the reaction proceeds the samples were dissolved in D_2O neutralized to pH 7 by the addition of NaOD. The collected ^{1}H-NMR spectra (Figure S9-11) reveal different decomposition kinetics:

- Fructose: the signals of the reagent disappears within the first 60 min leading to a silent spectrum which can be a symptom of the formation of material with extended graphitic domains.
- Citric acid: the signals of the reagent disappears within the first 30 min in favour of the formation of light molecular compounds (see ref. 1 for the full characterization of some of the observed molecules) as precursors towards the formation of the nanoparticles. Silent ^{1}H-NMR spectrum is now observed after 4 hours of reactions.
- Glucose: the 1H-NMR analysis still reveals the presence of reagent signals after 4 h of reaction.

Therefore, the thermal decomposition of the reagents followed the order fructose >> citric acid > glucose.

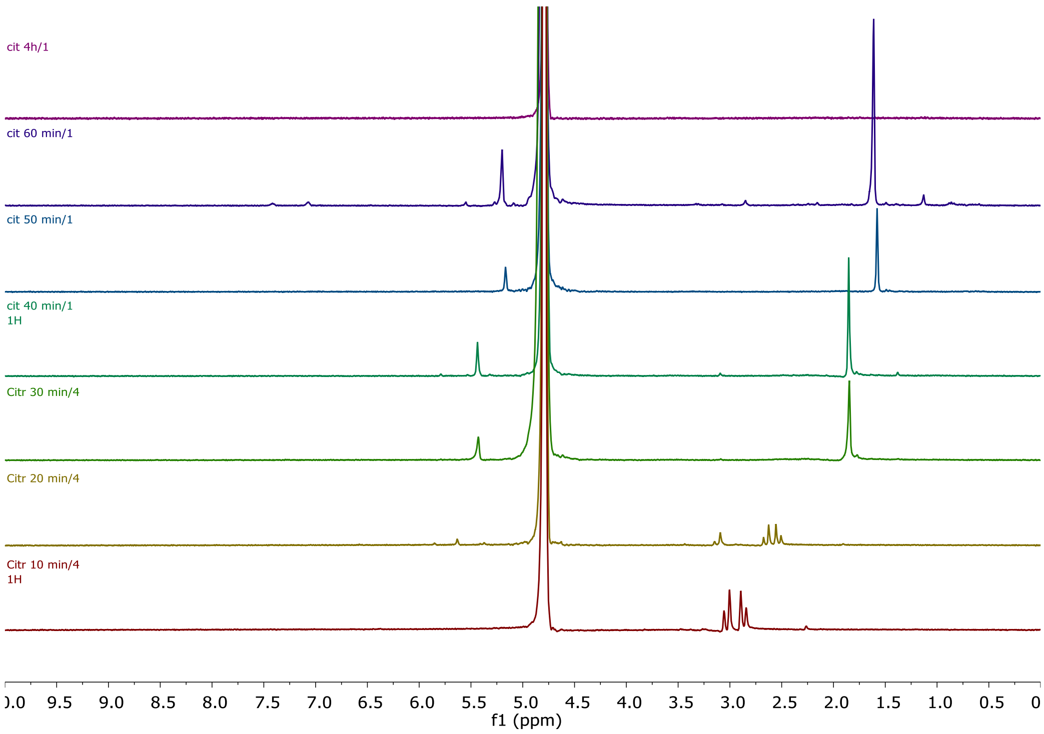

Figure S9. ^{1}H-NMR (D_2O) spectra for the thermal decomposition of citric acid.

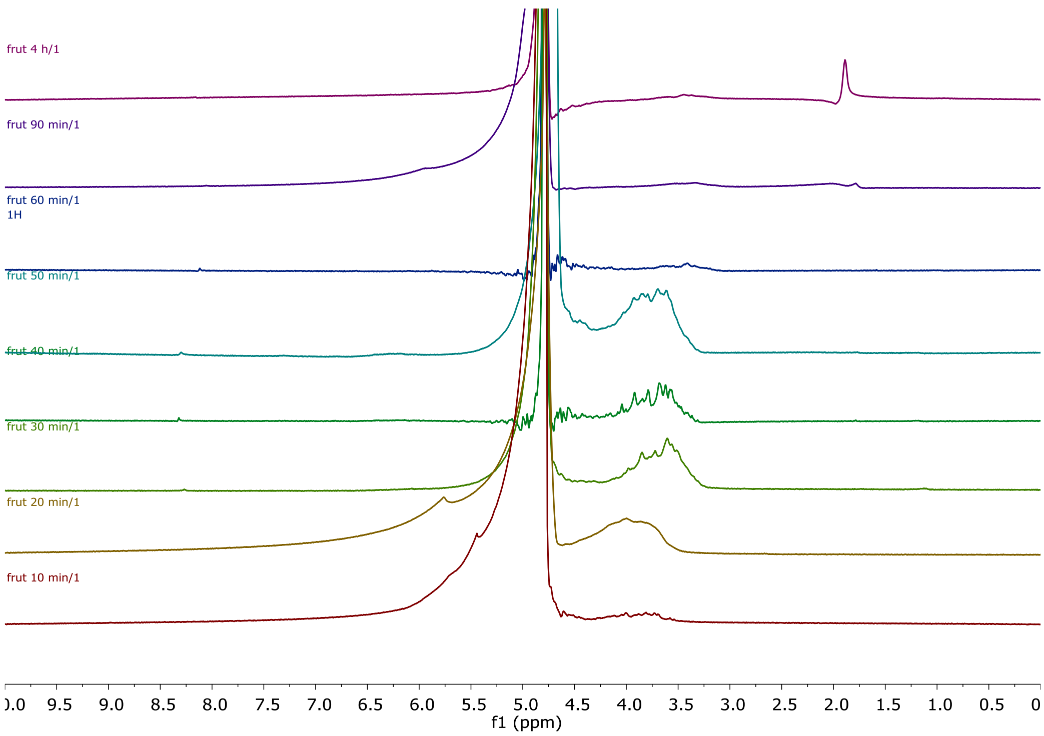

Figure S10. ^{1}H-NMR (D_2O) spectra for the thermal decomposition of fructose.

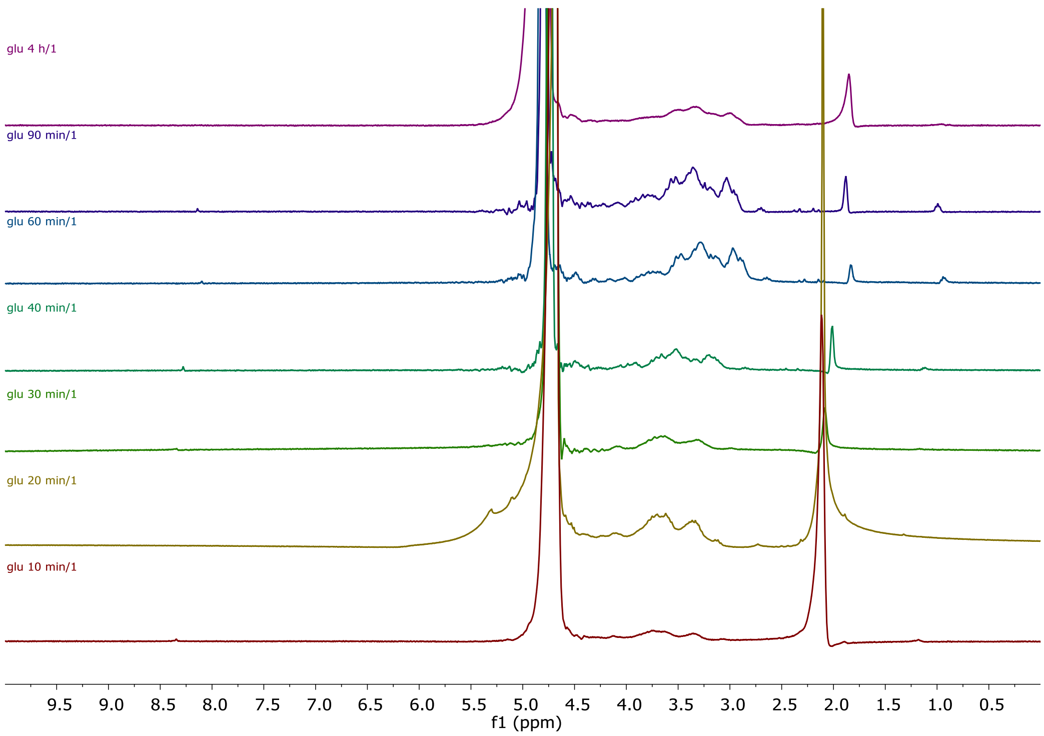

Figure S11. ^{1}H-NMR (D_2O) spectra for the thermal decomposition of glucose.

1.6. XPS analysis

X-ray photoelectron spectroscopy (XPS) was performed using a Perkin Elmer Φ 5600ci spectrometer using nonmonochromatic Al Kα radiation (1486.6 eV) in the 10−7 Pa pressure range. The binding energy (BE) values are referred to the Fermi level. The calibration of the BE scale was verified by checking the position of both Au4f7/2 and Cu2p3/2 bands (from pure metal targets), falling at 84.0 and 932.6 eV, respectively. After a Shirley-type background subtraction, the raw spectra were fitted using a nonlinear least-squares fitting program adopting Gaussian−Lorentzian peak shapes for all the peaks. Samples presented different shifts of all bands toward higher BEs as a consequence of surface charging: it was carefully determined and corrected in two steps: i) by using an internal reference (the C1s position of the band related to C=C bonds); ii) by checking, after the charging correction, the consistency of the BE positions of all the components evidenced in all the different XPS peaks. The final uncertainty of the determined BEs was not larger than 0.2 eV. The atomic composition was evaluated using sensitivity factors as provided by Φ V5.4A software. The relative uncertainty of the atomic fraction of the different elements is lower than 0.1.

1.7. FT-IR Spectra of CDs

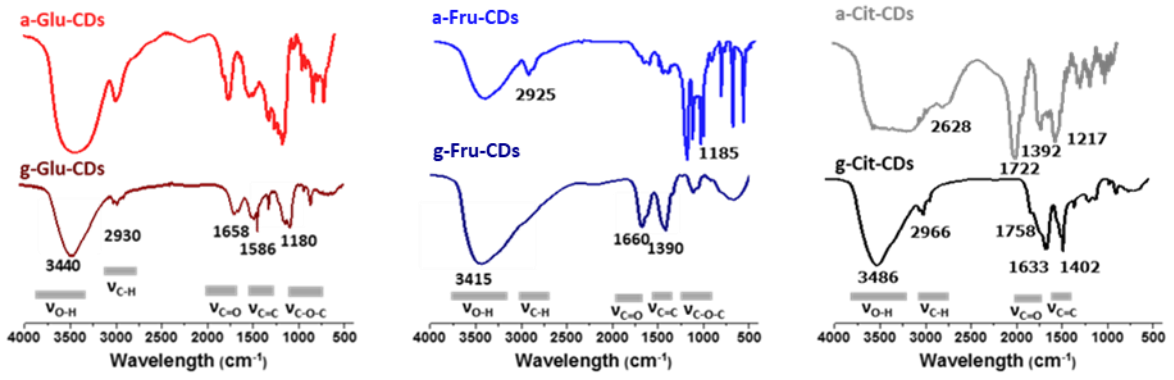

Figure S12. IR spectra of the amorphous and graphitic CDs.

1.8. UV-Vis spectra

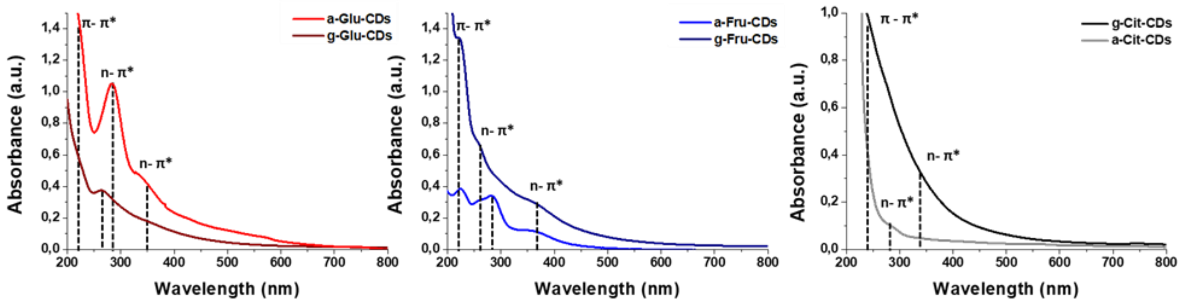

Figure S13. UV-Vis spectra of amorphous and graphitic CDs.

1.9. PLE spectra

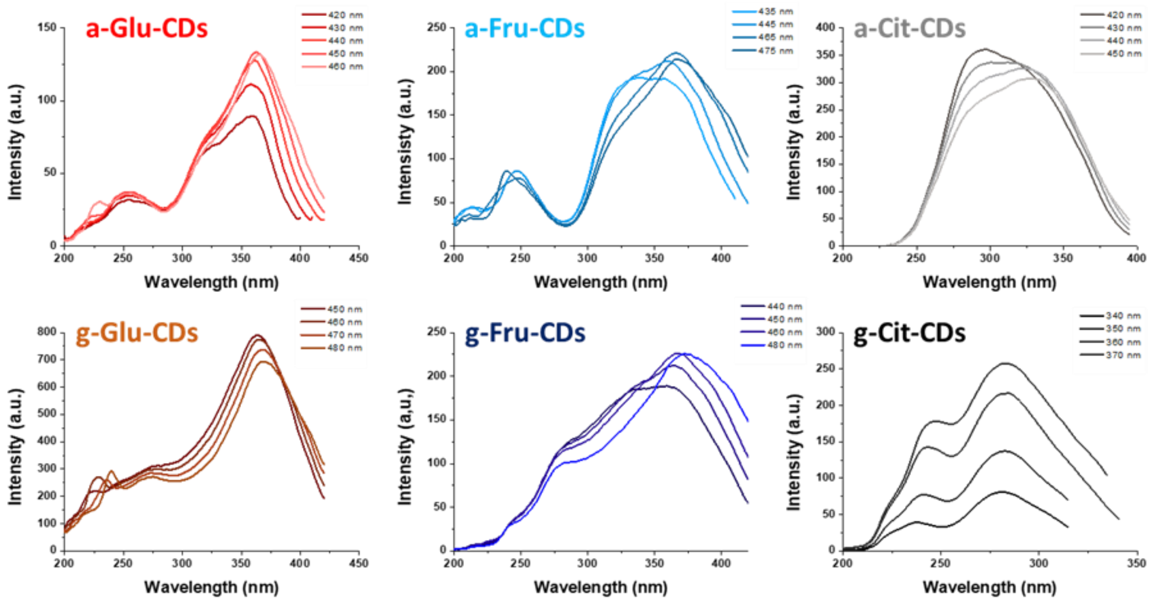

Figure S14. PLE spectra of graphitic and amorphous CDs

1.10. Time-resolved photoluminescence (PL) measurements

The time-resolved photoluminescence measurements showed a decay which is multi exponential. Therefore, they were fitted by using a triple-exponential curve and the resulting parameters are reported in Table S1.

Table S1. The average lifetime has been calculated as follows: $\langle\tau\rangle = \frac{A_1\tau_1^2 + A_2\tau_2^2 + A_3\tau_3^2}{A_1\tau_1 + A_2\tau_2 + A_3\tau_3}$

Entry	A1	t1 (ns)	A2	t2 (ns)	A3	t3 (ns)	< t > (ns)
a-Cit-CDs	0.31	0.78	0.32	1.25	0.34	4.26	**3.27**
g-Cit-CDs	0.23	0.19	0.45	0.72	0.17	6.87	**5.35**
a-Fru-CDs	0.34	0.31	0.35	1.25	0.14	6.63	**4.60**
g-Fru-CDs	0.87	0.59	0.05	1.63	0.09	4.79	**2.43**
a-Glu-CDs	0.39	0.49	0.39	0.64	0.10	6.79	**4.36**
g-Glu-CDs	0.28	0.36	0.37	0.80	0.21	7.25	**5.87**

1.11. Photocatalytic experiments

A solution composed by 0.1M of EDTA, 60 μM of MV^{2+} with an absorbance-normalized amount of CDs (a.b.s. = 0.5; 1.2 mg **a-Cit-CDs**, 0.21 mg **g-Cit-CDs**, 0.75 mg **a-Glu-CDs**, 1.5 mg **g-Glu-CDs**, 0.75 mg **a,g-Fru-CDs**) were placed under inert atmosphere in a quartz cuvette. The solutions were then irradiated at 365 nm (Hangar s.r.l.; ATON LED-UV 365; 80 W/m^2 of irradiance in the UV-A spectral range 315-400 nm). The progress of the reactions was monitored using an UV spectrophotometer following the formation of the typical absorption band of the reduced $MV^{\bullet+}$ radical cation species centered at 605 nm and its concentration was estimated using ε = 13700 $M^{-1}\cdot cm^{-1}$.[6]

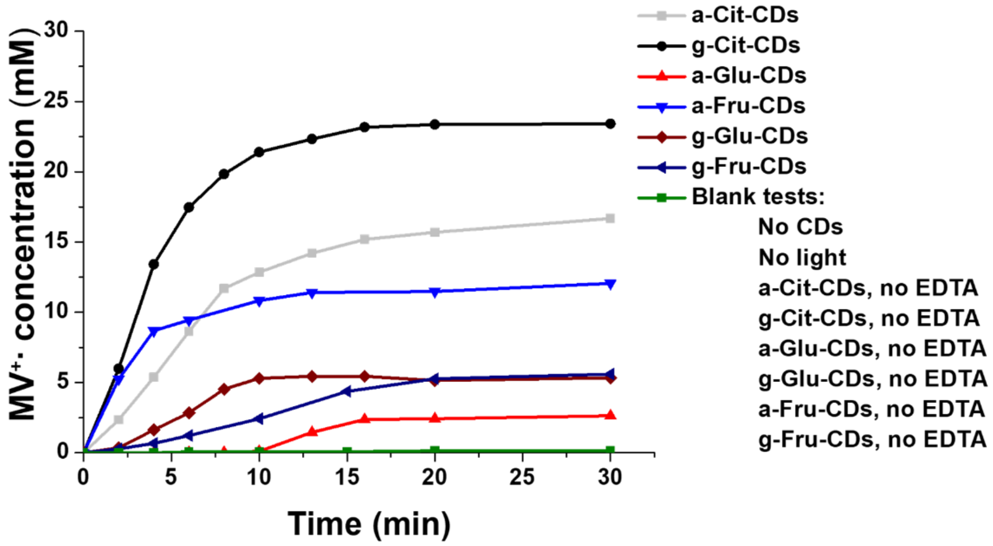

Figure S15. Evolution of reaction with CDs, MV^{2+} (60 μM) and EDTA (0.1M) under inert atmosphere at different irradiation time (λex = 365 nm).

1.12. References

[1] Cailotto, S.; Mazzaro, R.; Enrichi, F.; Vomiero, A.; Selva, M.; Cattaruzza, E.; Cristofori, D.; Amadio, E.; Perosa, A. *ACS Appl. Mater. Interfaces* **2018**, *10*, 40560-40567.

[2] Cailotto, S.; Amadio, E.; Facchin, M.; Selva, M.; Pontoglio, E.; Rizzolio, F.; Riello, P.; Toffoli, G.; Benedetti A.; Perosa, A. *ACS Med. Chem. Lett.* **2018**, *9*, 832-837.

[3] Meyer, J. C.; Geim, A. K.; Katsnelson, M. I.; Novoselov, K. S.; Booth T. J.; Roth, S. *Nature* **2007**, *446*, 60-63.

[4] Meyer, J. C.; Geim, A. K.; Katsnelson, M. I.; Novoselov, K. S.; Obergfell, D.; Roth, S.; Girit, C.; Zettl, A. *Solid State Commun.* **2007**, *143*, 101-109.

[5] Hernandez, Y.; Nicolosi, V.; Lotya, M.; Blighe, F. M.; Sun, Z.; De, S.; McGovern, I. T.; Holland, B.; Byrne, M.; Gun'Ko, Y. K.; Boland, J. J.; Niraj, P.; Duesberg, G.; Krishnamurthy, S.; Goodhue, R.; Hutchison, J.; Scardaci, V.; Ferrari, A. C.; Coleman, J. N. *Nat. Nanotechnol.* **2008**, *3*, 563-568.

[6] Striepe, L.; Baumgartner, T. *Chem. Eur. J.* **2017**, *23*, 16924-16940.
